# Integrating molecular point-of-care testing for influenza into primary care: a mixed-methods feasibility study

**DOI:** 10.3399/bjgp20X710897

**Published:** 2020-07-14

**Authors:** Simon de Lusignan, Uy Hoang, Harshana Liyanage, Manasa Tripathy, Ivelina Yonova, Rachel Byford, Filipa Ferreira, Javier Diez-Domingo, Tristan Clark

**Affiliations:** Nuffield Department of Primary Care Health Sciences, University of Oxford, Oxford, UK; Department of Clinical and Experimental Medicine, University of Surrey, Guildford, UK.; Nuffield Department of Primary Care Health Sciences, University of Oxford, Oxford, UK; Department of Clinical and Experimental Medicine, University of Surrey, Guildford, UK.; Nuffield Department of Primary Care Health Sciences, University of Oxford, Oxford, UK; Department of Clinical and Experimental Medicine, University of Surrey, Guildford, UK.; Nuffield Department of Primary Care Health Sciences, University of Oxford, Oxford, UK; Department of Clinical and Experimental Medicine, University of Surrey, Guildford, UK.; Nuffield Department of Primary Care Health Sciences, University of Oxford, Oxford, UK; Department of Clinical and Experimental Medicine, University of Surrey, Guildford, UK.; Nuffield Department of Primary Care Health Sciences, University of Oxford, Oxford, UK; Department of Clinical and Experimental Medicine, University of Surrey, Guildford, UK.; Nuffield Department of Primary Care Health Sciences, University of Oxford, Oxford, UK; Department of Clinical and Experimental Medicine, University of Surrey, Guildford, UK.; Vaccine Research Department, FISABIO-Public Health, Valencia, Spain.; Academic Unit of Clinical and Experimental Sciences, University of Southampton; National Institute for Health Research (NIHR) post-doctoral fellow, NIHR Southampton Biomedical Research Centre, Southampton, UK.

**Keywords:** antibiotic, antiviral, general practice, influenza, medical record systems, point-of-care systems

## Abstract

**Background:**

Molecular point-of-care testing (POCT) for influenza in primary care could influence clinical care and patient outcomes.

**Aim:**

To assess the feasibility of incorporating influenza POCT into general practice in England.

**Design and setting:**

A mixed-methods study conducted in six general practices that had not previously participated in respiratory virology sampling, which are part of the Royal College of General Practitioners Research and Surveillance Centre English sentinel surveillance network, from February 2019 to May 2019.

**Method:**

A sociotechnical perspective was adopted using the Public Health England POCT implementation toolkit and business process modelling notation to inform qualitative analysis. Quantitative data were collected about the number of samples taken, their representativeness, and the virology results obtained, comparing them with the rest of the sentinel system over the same weeks.

**Results:**

A total of 312 POCTs were performed; 276 were used for quantitative analysis, of which 60 were positive for influenza and 216 were negative. The average swabbing rate was 0.4 per 1000 population and swab positivity was between 16.7% (*n* = 14/84) and 41.4% (*n* = 12/29). Given a positive influenza POCT result, the odds ratio of receiving an antiviral was 14.1 (95% confidence intervals [CI] = 2.9 to 70.0, *P*<0.001) and of receiving an antibiotic was 0.4 (95% CI = 0.2 to 0.8, *P* = 0.01), compared with patients with a negative result. Qualitative analysis showed that it was feasible for practices to implement POCT, but there is considerable variation in the processes used.

**Conclusion:**

Testing for influenza using POCT is feasible in primary care and may improve antimicrobial use. However, further evidence from randomised trials of influenza POCT in general practice is needed.

## INTRODUCTION

Influenza is associated with high levels of morbidity and mortality.^1^ In the UK, influenza is estimated to account for 11.5% of all episodes of respiratory infection, with approximately 67 000 patients admitted to hospital and 2000 deaths per year.^2^ Vaccination is suboptimally effective at preventing influenza,^3^ and antivirals may improve clinical outcome, especially when administered early in the course of disease.^4^^,^^5^

Current antiviral treatment for influenza needs to be administered within 48 hours from onset of symptoms for optimal efficacy.^6^^,^^7^ A novel antiviral, baloxavir, has been shown to improve the time to resolution of symptoms and reduce complications in high-risk patients with influenza.^8^ Use of these new agents, once approved in the UK, will likely be restricted to patients with microbiologically confirmed diagnosis given their cost.^9^

In the last few years, highly accurate rapid molecular test platforms for influenza have become available.^10^ Point-of-care testing (POCT) for influenza has the potential to improve clinical decisions and patient outcomes as a result of a more appropriate use of antibiotics, antivirals, and infection control measures.^11^ Additionally, POCT could provide information to enhance influenza disease surveillance and clinical research, particularly providing data to compare vaccines in clinically important subgroups, and antiviral therapy in real-world trials.^12^

Public Health England (PHE) has produced advice for institutions interested in using influenza POCT (Supplementary Box S1).^13^

The aim of this study was to assess the feasibility of incorporating influenza POCT into general practice workflows and to explore its potential impact on antimicrobial use.

## METHOD

A mixed-methods, multi-site cohort study was used to investigate the implementation of influenza POCT in primary care workflows. The protocol for this study has previously been published.^14^

### Study setting and population

The study took place between February 2019 and May 2019, during the influenza season as defined by PHE. It was nested in the English national sentinel surveillance network run by the Royal College of General Practitioners (RCGP) Research and Surveillance Centre (RSC), one of the longest established primary care sentinel networks in Europe.^15^^,^^16^ Previous work has shown that the age and sex distribution of patients in the sentinel network is broadly similar to the English national census distribution.^15^

**Table:** How this fits in

Highly accurate rapid molecular test platforms for influenza have been evaluated in secondary care settings by Public Health England, but no data on their use in primary care settings in the UK have been published to date. This study, nested in six general practices that are part of the English national sentinel surveillance network, explored the feasibility and impact of implementing molecular point-of-care testing (POCT) in primary care. The study showed an impact on antibiotic and antiviral use: patients with a positive POCT test were significantly less likely to be prescribed an antibiotic and significantly more likely to be prescribed an antiviral medication. The study results are helpful for healthcare providers, commissioners, and policymakers interested in the use of POCT to monitor influenza in primary care and its impact on the clinical care of patients.

Six practices with a registered population of approximately 78 500 patients took part in the study. These practices had not previously participated in respiratory virology swabbing, according to RCGP RSC records, and were considered as ‘naive’ to respiratory virus sampling. The Abbott ID Now POCT machine was used for this study. Its diagnostic accuracy has previously been assessed: in a systematic review published in 2017, Merckx *et al* ^10^ reported a sensitivity in adults of 80.3% (95% confidence intervals [CI] = 63.7 to 90.8%) and 68.5% (95% CI = 40.2 to 87.2%) for influenza A and B, respectively. Vos *et al* ^17^ reported a pooled sensitivity of 81.6% (95% CI = 75.4 to 87.9%) for respiratory viruses and pooled specificity of 94.0% (95% CI = 86.0 to 100%). This compares with a pooled sensitivity of all rapid molecular respiratory viral tests of 90.9% (95% CI = 88.7 to 93.1%) and pooled specificity of 96.1% (95% CI = 94.2 to 97.9%).^17^ At the time of the study, key advantages to using the Abbott ID Now test over alternatives (such as the Roche Cobas Liat test) in primary care were that the consumables could be stored at room temperature and did not require additional cold storage space, and that the test was quicker, which is important in a busy primary care clinic setting. Another key advantage of the Abbott test system is the overall cost of machine and consumables, which are substantially less than the Roche test system, at the time of the study. Clinicians in the practices were encouraged to undertake influenza POCT swabs from consented patients presenting with an acute influenza-like illness and acute respiratory illness, during the weeks when the sentinel network suggested that influenza was circulating. Practices were provided with a leaflet explaining the study to staff and eligible patients, and were encouraged to display a poster about the study in their waiting areas.

### Data collection and analysis

A sequential explanatory approach was taken to data collection in this study,^18^ with quantitative data analysis followed by qualitative interviews with the study’s practice participants.

The primary quantitative outcome was the number of valid influenza swabs taken and tested by the study practices. These data were collected from POCT machines. The virology results obtained from study practices and the representativeness of swabbed populations were compared with the rest of the sentinel system over the same weeks.

Invalid or voided swabs were those swabs that did not provide a negative or positive influenza result or were swabs that had been used for the purposes of quality assurance or staff training. For the purposes of this study, swabs that were performed on patients who had registered to opt out of data sharing as per the national data opt-out policy,^19^ with no medical record being made available to anyone outside their direct patient care, were also considered void.

Secondary quantitative outcomes, including age, sex, socioeconomic status, ethnicity, chronic conditions, influenza vaccination status, antibiotic prescribing, influenza antiviral prescribing, and influenza vaccine effectiveness, were obtained by linking the results from POCT machines to details from the patient’s electronic medical record, as outlined in a previously published protocol.^14^ Socioeconomic status was measured using the Index of Multiple Deprivation (IMD) score.^20^

Secondary qualitative outcomes on the implementation of the POCT machines were collected using a semi-structured questionnaire survey of practice staff (Supplementary Box S2). Responses from the questionnaires were reviewed to elicit common themes related to the following domains, which have previously been shown to be important determinants of implementation of influenza POCT in clinical practice: performance of the POCT platform; clinical pathways and staff training; result reporting; clinical governance; costs; and monitoring of effectiveness.^13^

A sociotechnical perspective informed the assessment of qualitative information from the questionnaires. This research outlook focuses on the interdependence and inextricable linkages between people and technological systems.^21^ The authors compared business process models within each practice to assess which were successful at integrating POCT into their workflows, thus able to jointly optimise their sociological and technological systems to produce positive outcomes. Business process models are graphical representations of the commercial and organisational workflow processes within an organisation. This is helpful to model collaborations and business transactions within health systems. Business processes were modelled using the Business Process Modelling Notation (BPMN).^22^ BPMN can be used to depict the end-to-end flow of a business process. The notation has been specifically designed to coordinate the sequence of processes and the messages that flow between different process participants in a related set of business activities.^23^

## RESULTS

**Figure 1.**
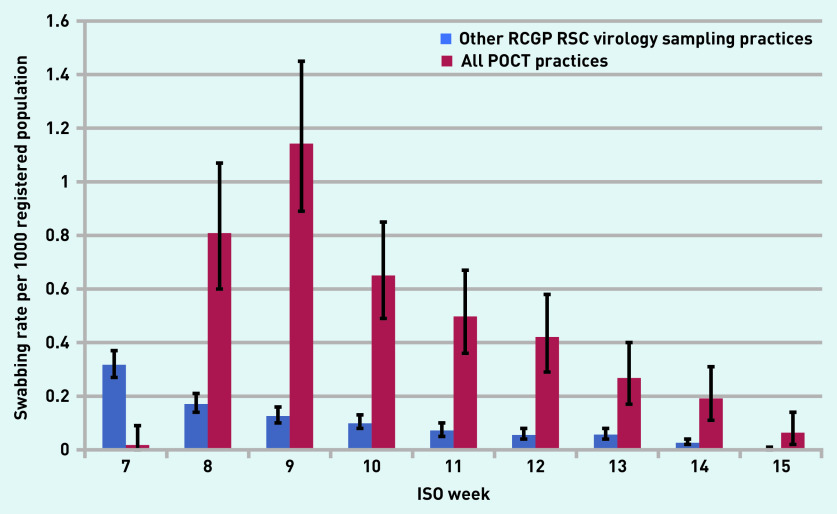
***Comparison of swabbing rates per 1000 registered population in all POCT practices versus other RCGP RSC virology sampling practices. The POCT practices conducted tests in-house while the RCGP RSC sentinel practices sent samples to the Public Health England reference laboratory. ISO = International Organization for Standardization. POCT = point-of-care test. RCGP RSC = Royal College of General Practitioners Research and Surveillance Centre.***

**Figure 2.**
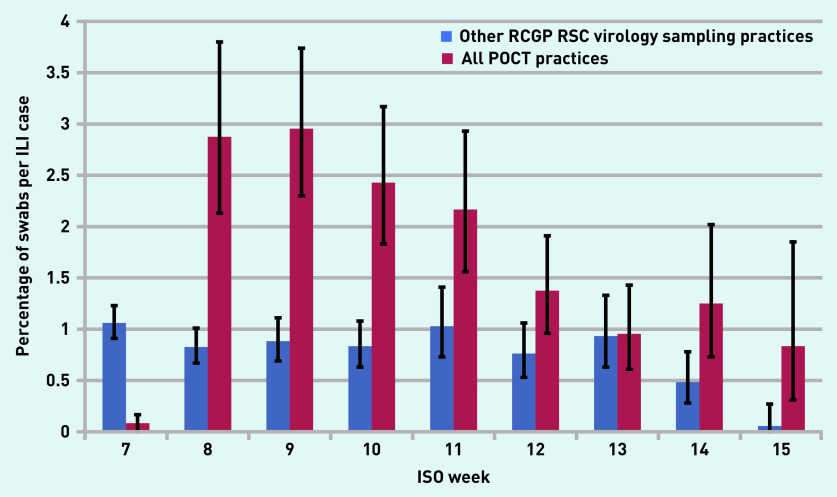
***Comparison of swabbing rates and percentage of ILI cases in all POCT practices compared with the RCGP RSC sentinel virology sampling practices. ILI = influenza-like illness. ISO = International Organization for Standardization. POCT = point-of-care test. RCGP RSC = Royal College of General Practitioners Research and Surveillance Centre.***

In total, 312 tests were recorded by the POCT machines. This equates to approximately six swabs per practice per week over the duration of the study. This compares favourably with the rate of influenza swabbing in the rest of the sentinel network of just under one swab per practice per week. Figures 1 and 2 show that POCT practices performed more swabs than the other RCGP RSC virology sampling practices when their practice population size and influenza-like illness rates were taken into account. The average swabbing rate for POCT practices was 0.4 compared with 0.1 per 1000 population for other RCGP RSC virology sampling practices (*P* = 0.15).

After cross-checking data from the POCT machines with electronic medical records from the practices, 36 tests were deemed to be void. In total, 276 swab results were used for the quantitative analysis, of which 60 were positive for influenza and 216 were negative. There was substantial variation in the number of swabs taken between practices over the course of the study (Supplementary Figures S1–S3).

Practice F had the highest swabbing rates of 2.8 per 1000 population taking into account its practice size. However, taking into account the number of influenza-like illness case presentations, practice A had the highest swabbing rate with 15 swabs per influenza-like illness case presentation in International Organization for Standardization week 9.

Of the six study practices, 59.8% (*n* = 165/276) of swabs taken were in females (Supplementary Figure S4), this compares with data from other RCGP RSC virology sampling practices where 58.0% of swabs were taken in females. More swabs were undertaken in patients <20 years of age, although individual practices varied in the age distribution of the patients that they swabbed. The proportion swabbed from each age group compared favourably with the swabbing rate by age for other RCGP RSC virology sampling practices (Supplementary Figure S5). More swabs were undertaken in patients in the most deprived quintiles (Supplementary Figure S6). A total of 25 of 276 (9.1%) swabs were taken from patients of black or ethnic minority origin (Supplementary Figure S7), which is slightly lower than the proportion of patients of black or ethnic minority origin registered with study practices or presenting with influenza-like illness (approximately 13.1%; data not shown).

**Figure 3.**
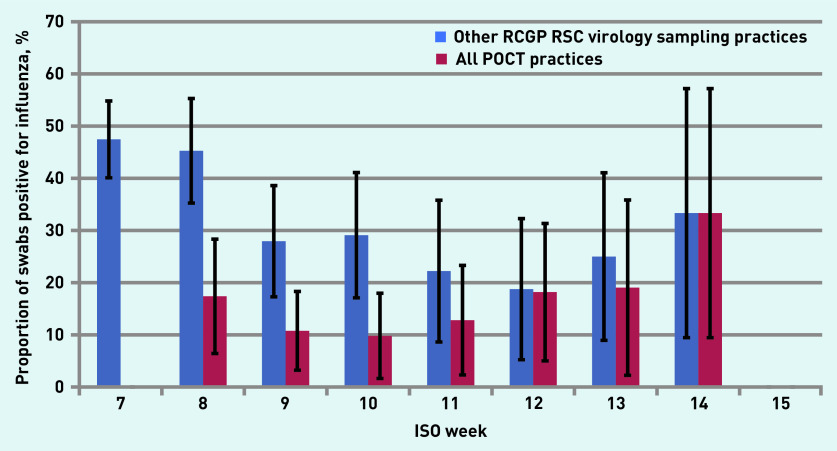
***Swab positivity rate for influenza in POCT practices versus other RCGP RSC virology sampling practices. No positive swabs were taken in ISO week 15. ISO = International Organization for Standardization. POCT = point-of-care test. RCGP RSC = Royal College of General Practitioners Research and Surveillance Centre.***

Practices varied two-fold, between 20.0% (*n* = 1/5) and 54.8% (*n* = 17/31), in the proportion of swabs taken from people with existing risk factors for influenza infection, as defined by the English Chief Medical Officer (Supplementary Figure S8).^24^ The overall positivity rate for swabs for influenza varied from 16.7% (*n* = 14/84) to 41.4% (*n* = 12/29) between practices (Supplementary Figure S9). Considered as a whole, the swab influenza positivity rate for practices that used POCT compared favourably with the positivity rate of swabs collected by other RCGP RSC virology sampling practices (Figure 3).

The proportion of invalid test results was low across all practices, with an average of 5.4% (*n* = 15/276) of tests yielding an invalid result (Supplementary Figures S9 and S10).

Using data about vaccination status of patients who were swabbed showed that the odds ratio (OR)^25^ for influenza vaccine effectiveness was 1.1 (95% CI = 0.6 to 2.0) and the risk ratio for influenza in those who have been vaccinated was 1.1 (95% CI = 0.7 to 1.7), although the small sample size makes the interpretation of results difficult (Supplementary Table S1).

**Figure 4.**
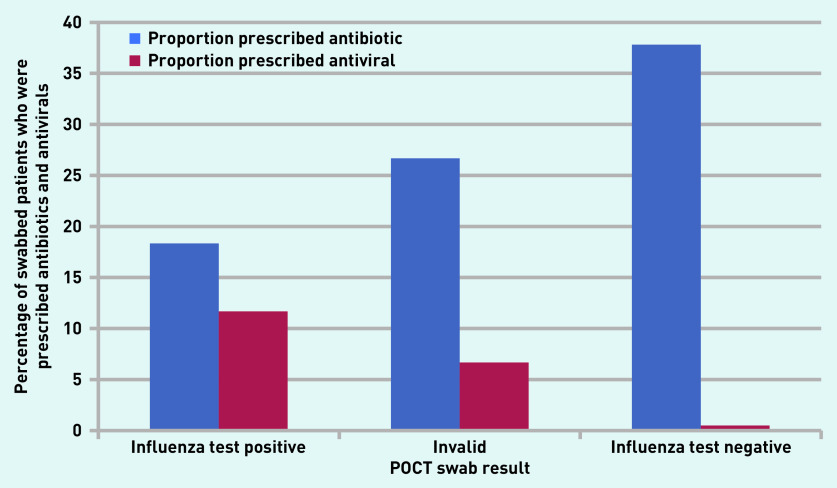
***Proportion of swabbed patients who received antibiotics or antivirals following influenza point-of-care testing. POCT = point-of-care test.***

Using data about antibiotic and antiviral prescribing for patients who were tested showed that 37.8% (*n* = 76/201) of patients received antibiotics following a negative influenza POCT result (Figure 4). The OR for being prescribed an antibiotic given a positive result was 0.4 (95% CI = 0.2 to 0.8; *P* = 0.01) compared with a negative test (Supplementary Table S2). Conversely, the OR for receiving an antiviral given a positive result was 14.1 (95% CI = 2.9 to 70.0; *P*<0.001) compared with a negative test. The OR for being prescribed an antiviral in those testing positive for influenza and with an existing condition identified by the Chief Medical Officer as at high risk of complications from influenza infection was 16.0 (95% CI = 2.9 to 88.9; *P*<0.001) (Supplementary Tables S3 and S4).^24^

**Figure 5.**
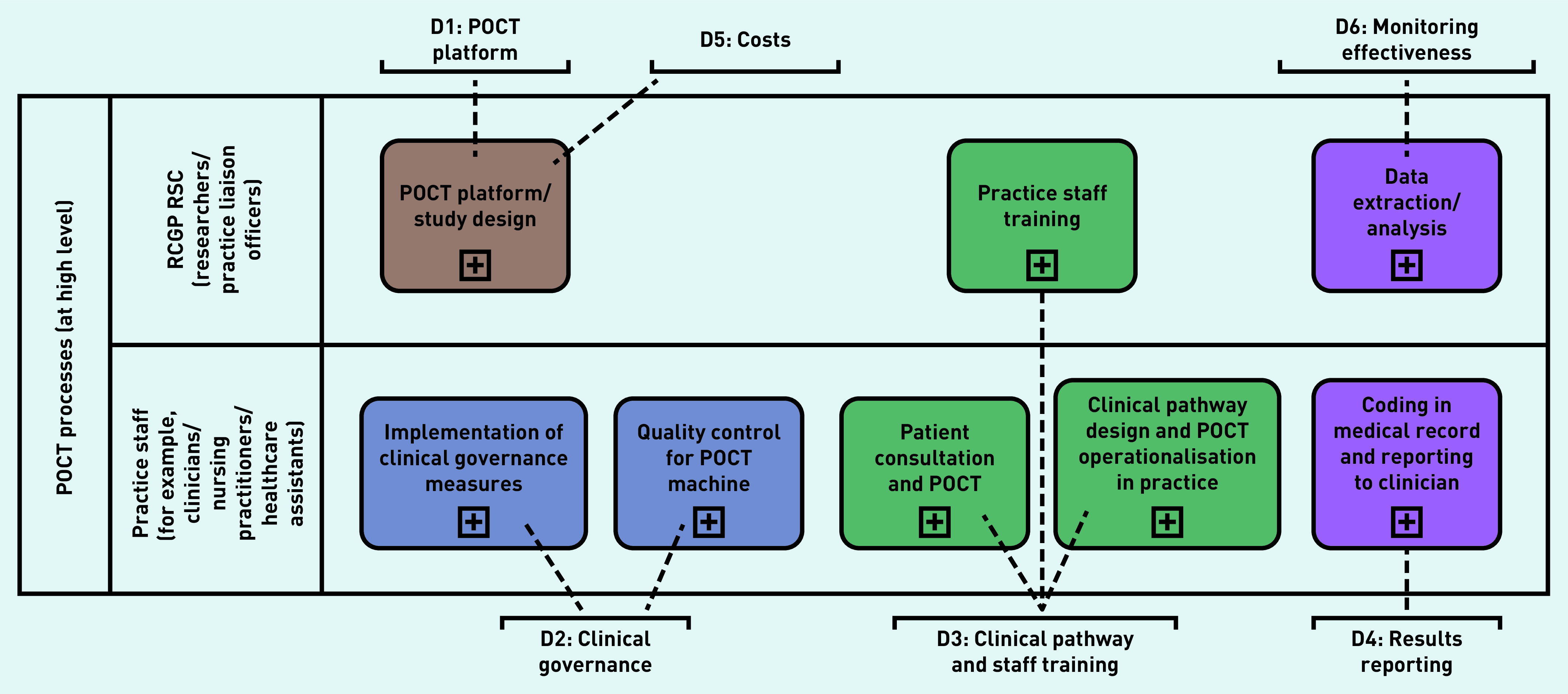
***Sub-processes identified during the qualitative evaluation mapped to the six domains of the Public Health England POCT implementation checklist. D = domain. POCT = point-of-care test. RCGP RSC = Royal College of General Practitioners Research and Surveillance Centre.***

Qualitative analysis showed considerable variation in implementing POCT processes in the participating practices (Table 1). At a high level, common sub-processes that could be mapped to the PHE POCT implementation checklist were identified (Figure 5). The largest difference was observed between practices in the methods used to systematically identify patients eligible for swabbing and in who managed the POCT machines. These variations were primarily influenced by the clinical governance (Domain [D]2) and clinical pathway domains (D3) (Supplementary Figure S11 and S12).

**Table 1.** Information extracted from the interviews mapped onto the domains considered for analysis

**Domain**	**Evaluation measure**	**Practice A**	**Practice B**	**Practice C**	**Practice D**	**Practice E**	**Practice F**
**POCT platform**	Platform location	Mobile trolley	Dedicated room	Dedicated room	Dedicated room	Mobile trolley	Desk in reception area

**Clinical pathway and staff training**	Training	Training provided by study investigator. Additional training organised locally by practice lead for study	Training provided by study investigator. Additional training organised locally by practice lead for study	Training provided by study investigator. Additional training organised locally by practice lead for study	Training provided by study investigator. Additional training organised locally by practice lead for study	Training provided by study investigator. Additional training organised locally by practice lead for study	Training provided by study investigator. Additional training organised locally by practice lead for study
	Clinical algorithm used for patient identification	Triaged and swab taken by nurse practitioners	Candidates identified by clinician and swabbed in consultation room. Swab taken to POCT room within 2 hours	Candidates selected during regular appointments	EMIS template developed locally that opens when eligible patient present	No specific method used	Candidates selected during regular appointments
	Dissemination of clinical algorithm	No specific dissemination	During practice clinical meeting	Email/face-to-face	Dissemination by being incorporated into the clinical system	Introduction email/weekly email reminders	Daily email reminders
	Home visits considered?	Yes	No	Yes	Yes	No	Yes
	Consent	Nurse practitioner	Clinician	Nurse practitioner/clinician	Clinician	Nurse manager	Clinician

**Result reporting**	Action of the results	Results coded in the medical record by nurse practitioner	POCT results coded in the medical record by nurse practitioner	Results coded in the medical record by clinician/nurse practitioner	POCT results coded in the medical record by HCA	POCT results coded in the medical record by HCA. The lead has created a protocol with codes to standardised recording	POCT results coded in the medical record by HCA
	Results communication to clinician	POCT results are shared with the clinician	POCT results are shared with the clinician	POCT results are shared with the clinician	POCT results are shared with the clinician	POCT results are shared with the clinician	POCT results are shared with the clinician

**Clinical governance**	Role of practice lead for study	Nurse practitioner	Clinician	Clinician	Research administrator	Nurse manager	Practice manager
	Role of machine operator	Nurse	HCA	HCA	Research administrator	Practice manager	Practice manager
	Quality control	Nurse	HCA	HCA	Research administrator	Practice manager	Practice manager
	Stock supply management	Stock replenishment by machine operator	Stock replenishment by machine operator	Stock replenishment by machine operator	Stock replenishment by machine operator	Stock replenishment by machine operator	Stock replenishment by machine operator

**Costs**	Domain not assessed						

**Monitoring of** **effectiveness**		Planned to conduct local audit of POCT use	No plans for monitoring effectiveness	No plans for monitoring effectiveness	No plans for monitoring effectiveness	No plans for monitoring effectiveness	No plans for monitoring effectiveness

EMIS = Egton Medical Information Systems. HCA = healthcare assistant. POCT = point-of-care testing.

## DISCUSSION

### Summary

To the authors’ knowledge, this is the first time POCT for influenza has been robustly evaluated in general practice in the UK. This evaluation has shown that it is feasible to use the Abbott ID Now POCT machine to test for influenza in a sentinel network in primary care, with comparable overall swabbing and positivity rates for practices using POCT versus practices that participate in the usual virology sampling programme. However, there was a wide variation in swabbing rates between POCT practices.

The results of POCT also influenced clinical prescribing practices, with patients who received positive tests in primary care being significantly more likely to receive antivirals on the day of the swab result, especially if they were in the Chief Medical Officer’s risk groups for influenza,^24^ and those who received positive tests being significantly less likely to receive antibiotic prescriptions on the day of the swab result.

Qualitative analysis suggests that variations in implementation of the POCT platform in primary care were influenced by domains related to clinical governance of the machine and clinical pathways to testing. Higher swabbing rates were seen in practices with a clearly identified study lead or clinical champion with responsibility for the new technology. In addition, practices performed well if they had systematic methods for identifying suitable patients for swabbing, such as an electronic template mated to the patient’s electronic medical record.

### Strengths and limitations

Limitations of the study include the non-randomised nature of the study design, its short duration, and small sample size, because it was only possible to initiate the study halfway through the 2018/2019 influenza season. The POCT machines used did not include information about influenza subtype, which restricted interpretation of the results, especially those pertaining to the effectiveness of the seasonal influenza vaccine. Additionally, it was not possible to evaluate the full potential that POCT machines might have had if they had been used throughout the season when practice staff may have gained more experience with using the machines. Finally, information about the duration of respiratory illness was not collected before swab testing, thus this limits the conclusions regarding the appropriate use of antiviral medications following POCT.

Strengths of the study included that it was nested in the RCGP RSC English sentinel surveillance network, which allowed a comparison of the performance of practices using POCT for influenza testing versus practices that participate in the usual virology sampling programme conducted by PHE. The mixed methodology enabled the use of qualitative data from interviews with frontline staff to provide an understanding of some of the reasons for variations in the implementation of POCT between practices.

### Comparison with existing literature

The results of the current study are similar to those of a previous study, which suggested that clinicians are more likely to perform rapid testing for influenza compared with clinicians in control clinics using conventional centralised laboratory testing.^26^ The current study found that POCT practices performed up to six times more tests, although, after taking into account differences in influenza-like illness rates between practices, POCT practices swabbed at approximately double the rate of other RCGP RSC virology sampling practices. Gren *et al* ^26^ found that increased near-patient testing would result in alerts 9 days earlier than surveillance alerts via traditional systems. However, the small sample size and the late initiation of the current study preclude the ability to study this.

The results regarding the clinical impact of POCT contrast with a 2019 systematic review and meta-analysis of influenza POCT in ambulatory care, which suggested that POCTs had no effect on antibiotic prescribing rates (relative risk [RR] = 0.97, 95% CI = 0.82 to 1.15; *I*^2^ = 70%).^27^ However, in common with the study by Lee *et al*,^27^ the current study showed increased prescribing of appropriate antivirals for influenza (RR = 2.65; 95% CI = 1.95 to 3.60; *I*^2^ = 0%). The differences in the effects on antibiotic prescribing between the studies may relate to differences in study characteristics between the current study and the studies included in the meta-analysis, which were mainly randomised trials performed in paediatric emergency departments. Of the non-randomised studies that reported on antibiotic prescribing, four out of five reported significant reductions, although there was strong evidence of statistical heterogeneity, possibly as a result of the pooling of results from primary care and emergency departments. It is also of note that all studies included in the meta-analysis used antigen-based POCT, whereas the current study used molecular POCT.

### Implications for research

To the authors’ knowledge, this study provides the first evidence for the use of POCT to monitor influenza in primary care in the UK; however, further robust evidence is required, especially from randomised trials of POCT in general practice. Further work is required to study the impact of POCT on important public health tasks for sentinel surveillance, including influenza notification as well as infection control, and to study the cost–benefits of POCT in UK general practice.^28^
